# For Medical Directors: Case Report of a Missed Wooden Foreign Body in the Forehead

**DOI:** 10.7759/cureus.38790

**Published:** 2023-05-09

**Authors:** Jack Keehn, Steven Goodfriend, Martin Wegman

**Affiliations:** 1 Emergency Medicine, HCA Florida Orange Park Hospital, Orange Park, USA

**Keywords:** traumatic wounds, medical malpractice, apology, missed diagnosis, foreign body

## Abstract

Traumatic wounds, often contaminated with foreign material, are a common presenting complaint in the emergency setting. Unfortunately, embedded foreign material can go initially undetected or not be fully removed, leading to morbidity and becoming a common cause of medical malpractice claims. Here, we present a case of a missed wooden foreign body, including associated risk factors, potential contributing cognitive errors, recommendations to avoid such errors, and finally, a description of the case’s resolution. In addition, we will present steps taken after the error was recognized that would provide a better understanding to the patient and entail a “blameless” education plan to the team of clinicians. Developing a sincere and authentic connection with the patient and their family after the unexpected outcome is crucial. Additionally, these cases are outstanding learning tools for the individual clinician, as well as, the rest of the providers if processed in a non-blaming and educational manner.

## Introduction

Contamination of a traumatic wound with foreign material is a common concern in the emergency setting. The risk of unrecognized, retained foreign material is particularly high for puncture wounds and other wounds which develop from deeply penetrating injuries. Other risk factors include lack of sensation of the foreign body, late presentation (>24 hours) after wound development, failure to explore the wound by the clinician, or failure to obtain imaging to assess for foreign material [1].

Likely owing to high prevalence and ease of retrospective diagnosis, missed retained foreign bodies are one of the most common reasons for litigation related to the emergency department care [2,3]. The most common complication of retained foreign material is local tissue reaction and infection. Delayed wound healing, as well as, tendon or nerve injury can also occur [4].

Here we present a case of a wooden foreign body in a forehead wound which went undetected during two separate emergency department visits. The case information is based on a review of the electronic medical record as well as interview of the patient’s mother and the clinicians responsible for providing care over the three emergency department contacts. Written informed consent was provided by the patient’s mother to permit disclosure of the case and the associated images.

## Case presentation

**Figure 1:**
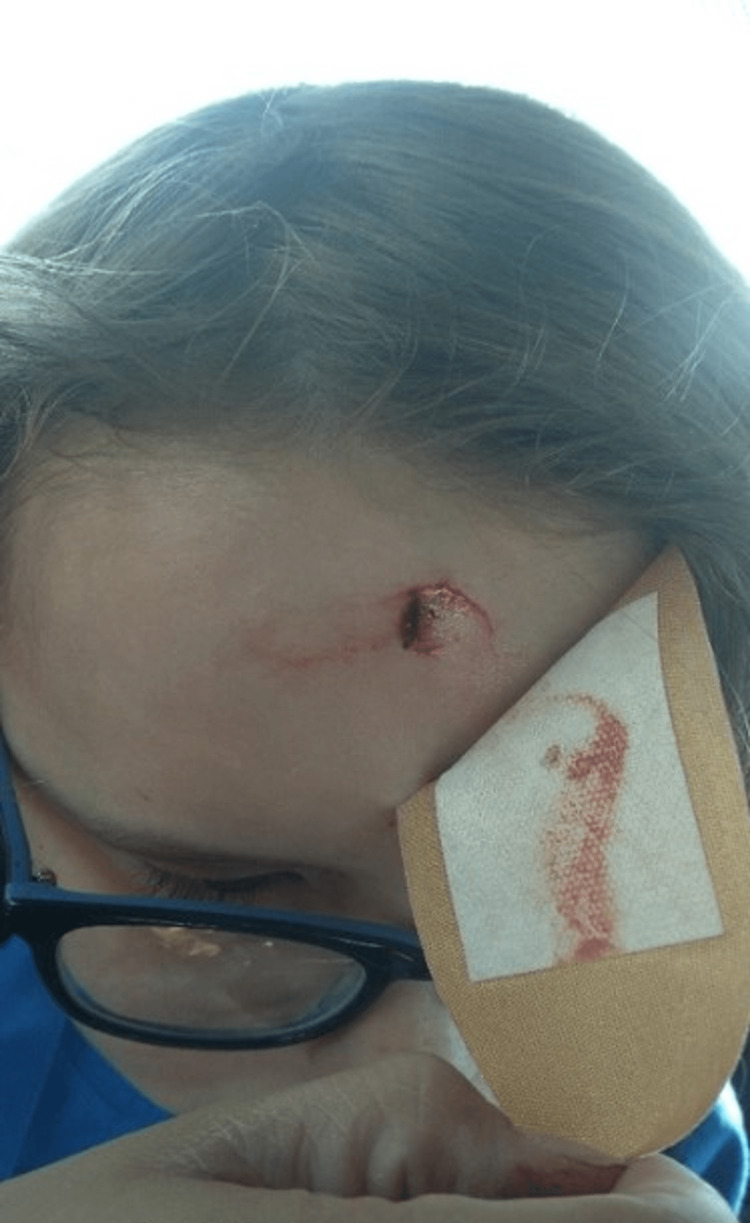
Initial wound

A previously healthy 10-year-old male presented to the emergency department with a forehead laceration which was sustained just prior to arrival. The patient reported that he swung a wooden stick at a tree and after striking the tree, the stick subsequently bounced backward, hitting his forehead. He denied loss of consciousness, headache, vision changes, eye pain, nausea, or vomiting. On exam, there was a hemostatic, superficial, linear, mid-superior forehead laceration approximately 1 cm in length (Figure 1). The edges were well approximated and there was no surrounding ecchymosis or hematoma. There was no palpable skull fracture or bony abnormality. The wound was explored for foreign bodies by gross visualization, but none were found. The laceration was then irrigated, cleaned, and closed with Dermabond tissue adhesive glue and steri strips. No imaging was performed, and the patient was discharged home.

Two days later, the patient returned to the same emergency department with his parents who were concerned due to increased swelling of his forehead and mild amount of purulent-appearing drainage from the wound. Reexamination by a second, different clinician revealed mild swelling to the forehead, associated tenderness and some serous exudate when the wound was expressed. The possibility of a retained foreign body was discussed with the patient’s father, but it was decided by the treating clinician that this was unlikely enough to not warrant re-exploration of the wound when weighed against increased discomfort, risk for infection, and size of scar formation. Again, no imaging was performed. The patient was discharged home on sulfamethoxazole/trimethoprim and cephalexin with a diagnosis of infected laceration. The family was agreeable with the plan.

**Figure 2:**
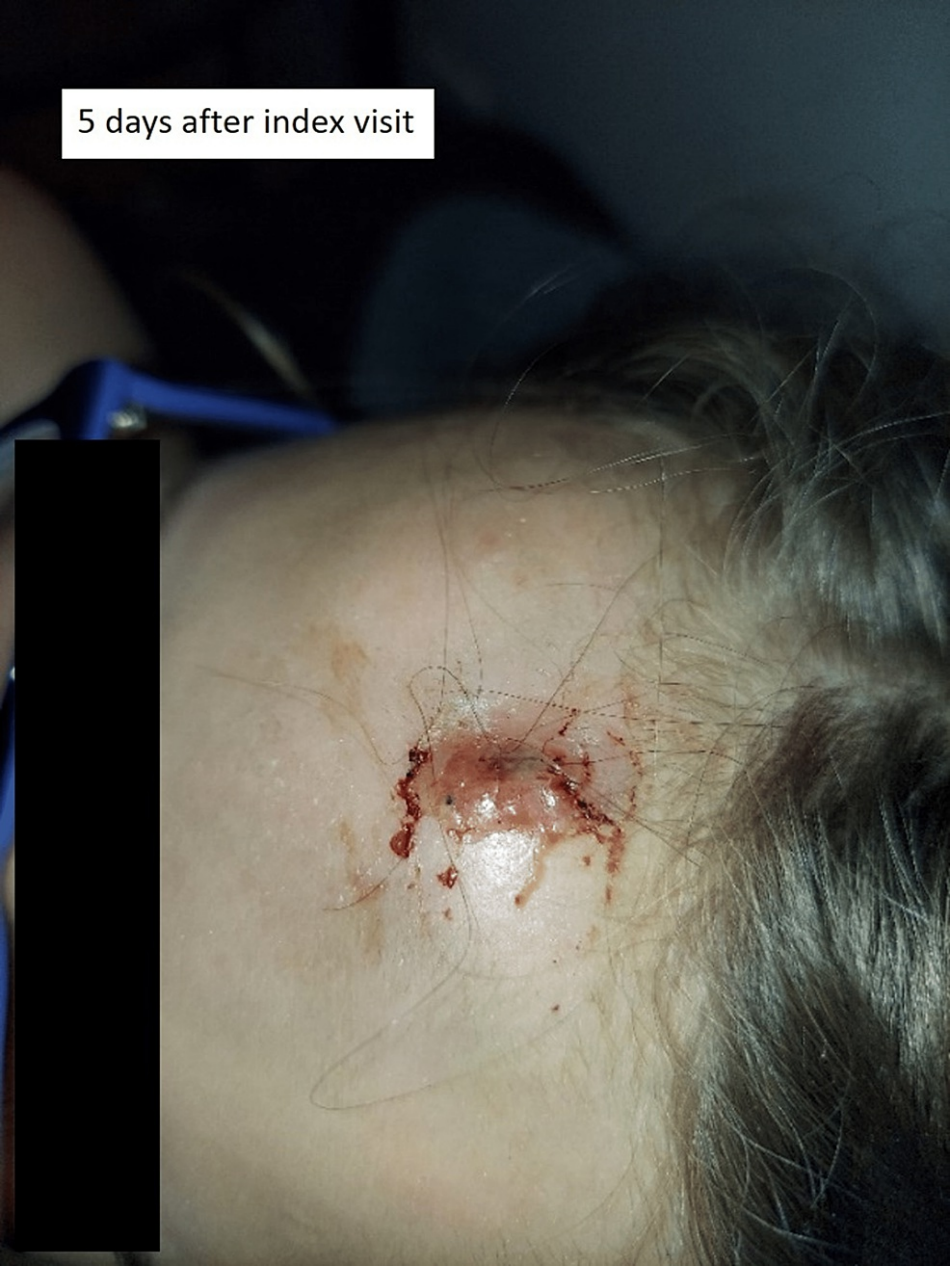
Infected wound

**Figure 3:**
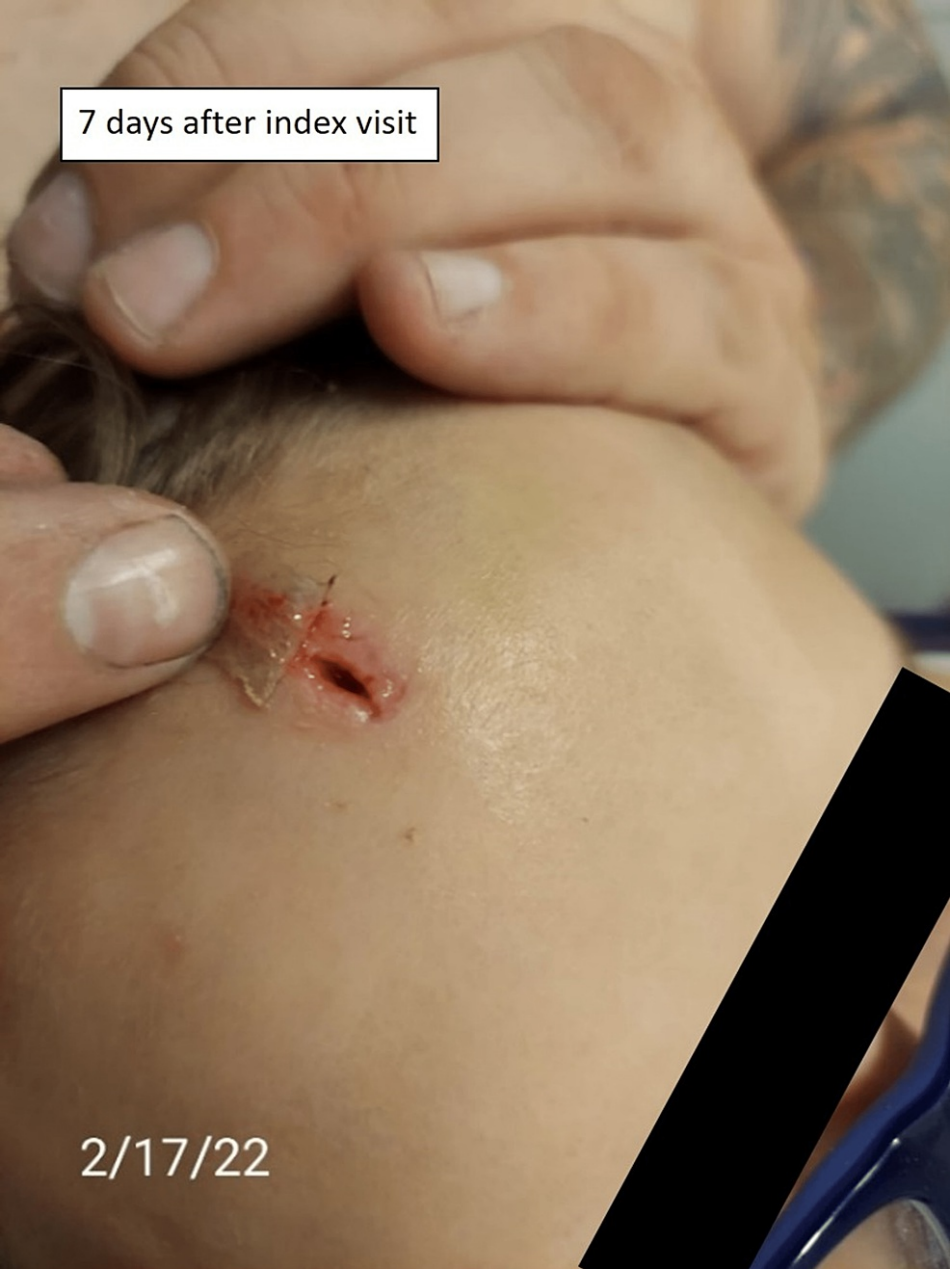
Post foreign body spontaneous expulsion

**Figure 4:**
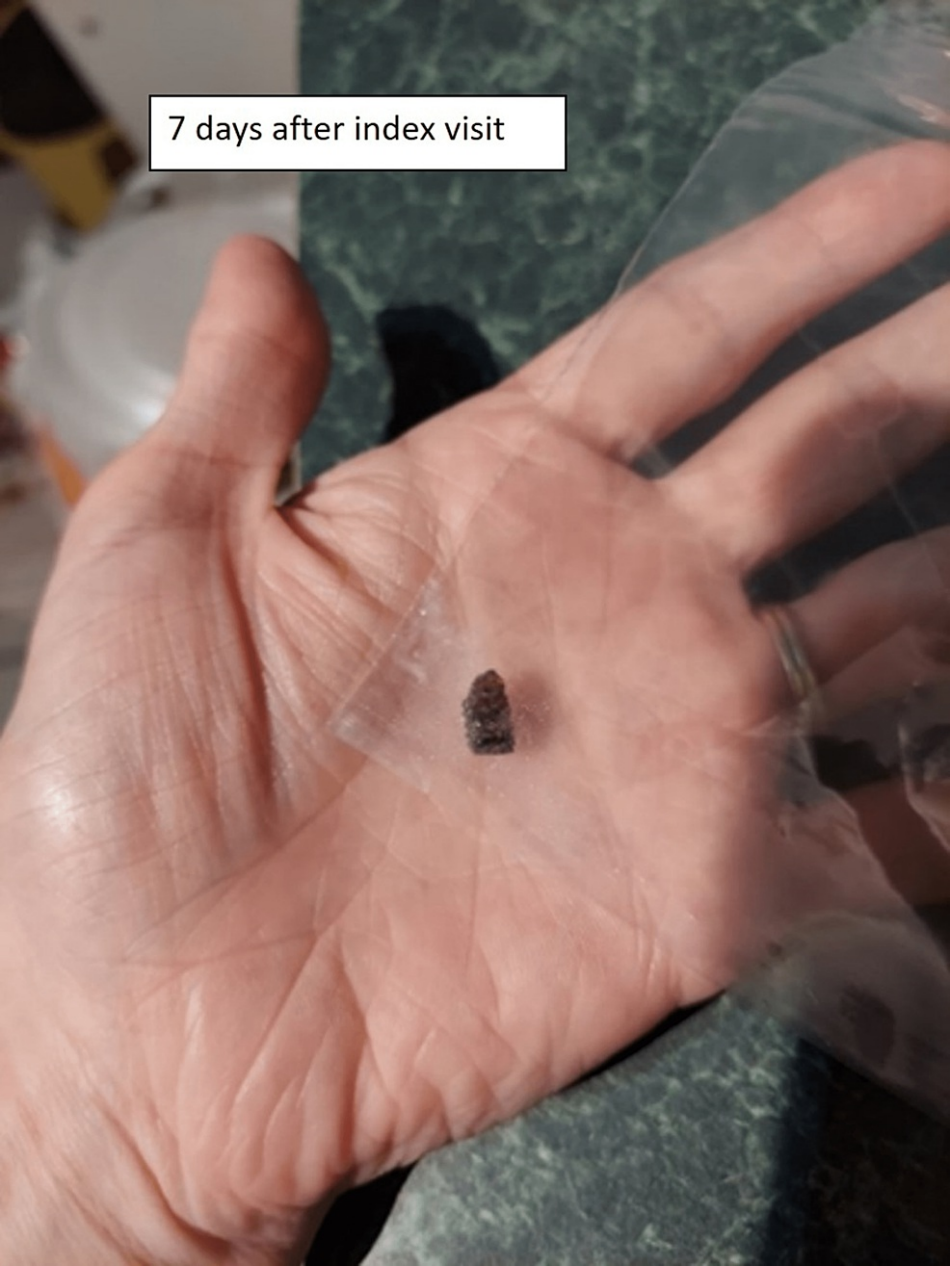
Wooden foreign body

Five days after the second emergency department visit, the parents posted an online complaint describing the situation with a picture of the wound (Figure 2). They also provided pictures of the wound after a wooden foreign body spontaneously emerged from the skin (Figure 3, Figure 4). Shortly thereafter the emergency department medical director reviewed the case and contacted the family to connect with them and get a better understanding of the situation.

The communication started with a sincere and heartfelt apology for the situation. It was then stated that, “We are grateful for you letting us know about this as it takes effort on your part, and these cases are usually great learning opportunities for the clinician and entire team. Without feedback, the team would not have awareness of an opportunity to improve.” It was also explained that we see over 100k patients a year, and it is hard to get every case perfectly correct, but we are committed to improving and getting better. “It's cases like these where we grow and learn and mitigate the risk of this happening again.” With these initial comments that illustrate a sincere desire to learn, the medical director proceeded to ask the parent to tell their story and their perspective. This involved active listening as well as note-taking to ensure all issues were heard and addressed.

In summary, the parents expressed frustration that the foreign body had not been detected or addressed during two separate emergency department visits. The shared facts and emotions were validated by the medical director. There was no debate nor justification of the care rendered but only active listening and validation. At the end of the conversation, an action plan was discussed with the family which included a commitment to educating the clinicians involved but also making this a learning case for the rest of the emergency department. The goal was to give this case a purpose and make things better for future patients that come into the emergency department. The family was grateful for the connection and the ability to be heard. They were very grateful that this would help others. With a few follow-up calls, the family agreed to allow this case to be presented in a manner that would impact more clinicians than just at this one site and hence giving us permission to present to a bigger audience.

As detailed above, this case was discussed with the involved clinicians in a non-shame nor blaming manner, and the clinicians were all self-reflective of the case. Additionally, it was presented at a quality conference, so that all of the clinicians could learn from this case, as it is a very common type of medical error. The case was very well received by the clinicians, and much learning and discussion ensued.

## Discussion

The above case describes a forehead wound with a missed foreign body, subsequent emergency department revisit, and ultimate case resolution. This case highlights several aspects of the history and physical exam important in evaluating wounds which may contain foreign bodies. For example, the type of foreign material exposure (e.g., glass, metal, wood) and the mechanism of injury can help inform imaging decisions or the clinician’s index of suspicion. Certain organic materials, such as wood, are more likely to become infected, and a high-velocity mechanism might force foreign bodies deeper through tissue. Physical exam should seek to exclude signs of injection (e.g., purulent drainage and increasing warmth, redness or pain) and include careful palpation and visualization, especially on a repeat visit. Depending on the affected areas, one should also assess neurological, vascular, and tendon function. Suspicion for retained foreign body is increased for penetrating wounds, other wounds that cannot be adequately visualized on gross exploration, wounds presenting in the setting of a missing portion of the striking object, and those caused by broken glass. In situations where there is a high index of suspicion but a foreign body is not detected, imaging should be strongly considered.

Multiple imaging modalities can be utilized to detect soft tissue foreign bodies. Most commonly, X-ray, computed tomography (CT), ultrasound, and MRI are used. A good initial study would be ultrasound due to how fast it can be performed and the low cost. Studies demonstrate CT and plain radiographs are very specific (98% for CT and 100% for radiographs) but are not very sensitive for detecting foreign bodies (63% for CT and 29% for radiographs); sensitivity and specificity of ultrasound for detecting foreign bodies is 72% and 92% [5,6]. If a trained provider is performing the ultrasound, the sensitivity for localizing foreign bodies can reach up to 96-100% [6]. If there is enough suspicion for organic foreign bodies (e.g., wood or thorns) which cannot be visualized on X-ray (which is better for identifying nonorganic compounds such as glass or plastic), ultrasound can better detect these types of foreign bodies, both radiolucent and radiopaque [6]. Therefore, most ED physicians, if trained in ultrasound, should use ultrasound as a first step in detecting foreign bodies.

This case provides potential examples of several types of cognitive bias. Infamously, it has been said that, “the most common cause of a misdiagnosis is a prior diagnosis.” This saying describes the phenomenon of anchoring bias, which occurs due to the propensity to rely too heavily on the first piece of information learned (Table 1) [7,8]. It is likely that the second treating clinician was subject to anchoring bias, particularly if they relied on the first clinician’s report that the wound was explored for foreign bodies, but none were found.

**Table 1:** Different types of cognitive bias

Cognitive bias type	Description
Availability bias	To think of things that come to mind easily as being more representative than reality
Premature closure	Tendency to stop thinking about further possibilities after making a diagnosis
Anchoring bias	Overly relying on the first diagnostic impression

As an alternative to anchoring, return visits to the emergency department can suggest an initially incorrect or inadequate diagnosis and treatment plan and represent an opportunity to correct such initial errors. One strategy we employ on return visits is to initially cognitively disregard the diagnosis made during the first encounter and re-collect the initial history while obtaining interval history and repeating the physical exam. In particular, non-healing or infected lacerations due to puncture wounds should raise clinician suspicion for foreign bodies.

Premature closure (Table 1), the tendency to arrest further thought about possible explanations or diagnoses likely occurred in this case. Premature closure can be a consequence of anchoring (Table 1), which occurs when one overly relies on the first diagnostic impression [7,8]. It is important to avoid the trap of anchoring as it can lead to premature closure. Additionally, availability bias (Table 1) describes the error in attributing a more cognitively straightforward explanation as one that is more representative of reality [7,8]. This error likely occurred as the second treating clinician assumed that the wound infection was due to an infected laceration rather than from retained foreign material.

When medical cases lead to poor outcomes, clinicians often fear having a discussion with the involved patient and family. This silence can commonly lead to false information and assumptions from third parties who were not directly involved in the care. This can lead to animosity and anger. Research supports speaking with patients and apologizing [9], as occurred in this case. The patient’s parents were ultimately thankful and appreciated the communication. Contrary to previous legal guidance, apologies can actually diminish patients’ likelihood of litigation, and patients who do pursue claims are more likely to settle when there is an apology [9]. This apology can also help to restore trust and leads to improved understanding and empathy.

It is important to use cases like the one presented here to provide non-punitive education in an effort to prevent future recurrences. Criticizing the care provided by the involved clinicians is commonplace, but this does not prevent future recurrences. This case was used to educate the entire emergency department during the morbidity and mortality conference, which had a tremendous impact since there were over 30 emergency department providers present. In addition, it was very helpful and therapeutic to the family to know that this experience would provide significant benefit to clinicians via education and mitigating the occurrence of these events in the future. It provided some meaning and purpose of this event for the family and allowed them to understand the imperfections that exist in healthcare.

## Conclusions

When there is any clinical suspicion for foreign bodies due to puncture wounds, a detailed history and thorough physical exam are critical in evaluation. If a foreign body is missed, complications such as infection, delayed wound healing, or loss of function can occur. It is imperative to identify foreign bodies and properly refer patients for definitive treatment.

If imaging is performed, a combination of radiographs and ultrasound provide a specificity of 100% and sensitivity up to 100% with a trained provider. If a foreign body is missed and later recognized, it is important to consider connecting with the patient and consider apologizing. Using these cases as learning opportunities are extremely powerful and may decrease the risk of them happening in the future.
